# Comparative effectiveness of angiotensin-converting enzyme inhibitors versus angiotensin II receptor blockers for major renal outcomes in patients with diabetes: A 15-year cohort study

**DOI:** 10.1371/journal.pone.0177654

**Published:** 2017-05-15

**Authors:** Hon-Yen Wu, Chiao-Ling Peng, Pei-Chun Chen, Chih-Kang Chiang, Chee-Jen Chang, Jenq-Wen Huang, Yu-Sen Peng, Yu-Kang Tu, Tzong-Shinn Chu, Kuan-Yu Hung, Kuo-Liong Chien

**Affiliations:** 1 Department of Internal Medicine, Far Eastern Memorial Hospital, New Taipei City, Taiwan; 2 Department of Internal Medicine, National Taiwan University Hospital and College of Medicine, Taipei City, Taiwan; 3 Institute of Epidemiology and Preventive Medicine, College of Public Health, National Taiwan University, Taipei City, Taiwan; 4 Faculty of Medicine, School of Medicine, National Yang-Ming University, Taipei City, Taiwan; 5 Clinical Informatics & Medical Statistics Research Center, Chang Gung University, Taoyuan City, Taiwan; 6 Department of Internal Medicine, National Taiwan University Hospital Hsin-Chu Branch, Hsinchu City, Taiwan; The University of Tokyo, JAPAN

## Abstract

**Background:**

Angiotensin converting enzyme inhibitors (ACEIs) and angiotensin II receptor blockers (ARBs) are considered to have similar renoprotective effects; so far there has been no consensus about their priorities. This study aimed to compare ACEIs and ARBs for major renal outcomes and survival in a 15-year cohort of adults with diabetes.

**Methods:**

This study utilized Taiwan’s medical and pharmacy claims data in the Longitudinal Cohort of Diabetes Patients. The primary outcome was long-term dialysis, and secondary outcomes were hospitalization for acute kidney injury, hospitalization for hyperkalemia, all-cause death, cardiovascular death, and non-cardiovascular death. Cox proportional hazards models were used to estimate the hazard ratios (HRs) and 95% confidence intervals (CIs) for outcomes comparing ACEIs with ARBs. We conducted subgroup analyses and interaction tests among patients with different age and comorbid diseases.

**Results:**

A total of 34,043 patients received ACEIs and 23,772 patients received ARBs. No differences were found for primary or secondary outcomes in the main analyses. ACEIs showed significantly lower hazard than ARBs for long-term dialysis among patients with cardiovascular disease (HR 0.80, 95% CI 0.66–0.97, interaction *P* = 0.003) or chronic kidney disease (0.81, 0.71–0.93, interaction *P* = 0.001).

**Conclusions:**

Our analyses show similar effects of ACEIs and ARBs in patients with diabetes. However, ACEIs might provide additional renoprotective effects among patients who have cardiovascular disease or chronic kidney disease.

## Introduction

The development and progression of chronic kidney disease are closely interrelated to hypertension [1, 2], and aggressive blood pressure-lowering management is able to decrease the risk of decline in renal function among patients with diabetes [3–5]. Angiotensin converting enzyme inhibitors (ACEIs) and angiotensin II receptor blockers (ARBs) are the two major classes of drugs among renin-angiotensin system (RAS) inhibitors, and are considered to have superior cardiorenoprotective effects than other classes of blood pressure-lowering drugs [6–9]. Therefore, major guidelines in the relevant specialty suggest ACEIs or ARBs as the first line blood pressure-lowering treatments for patients with diabetes [10–13].

Unlike the mechanisms of ARBs, ACEIs do not completely block the RAS; but ACEIs reduce the degradation of bradykinin and are considered to provide additional renoprotective effects [14]. The ONgoing Telmisartan Alone and in combination with Ramipril Global Endpoint Trial (ONTARGET) study, the largest randomized clinical trial comparing an ACEI with an ARB, reported similar effects on major renal outcomes in a study population with one-third of patients had diabetes [15]. The ONTARGET study was designed to evaluate composite cardiovascular outcomes among high risk patients, but not powered to detect differences of major renal outcomes [16]; and the study participants were not randomized based on the presence of diabetes (37% prevalence) or diabetic kidney disease (19% prevalence). Interpretations by meta-analytical approaches are also restricted by the limited number and power of randomized clinical trials [17]. A well-designed observational study can provide adequate participants numbers and follow-up time so as to achieve sufficient power for differentiating effects between ACEIs and ARBs. A few cohort studies compared ACEIs with ARBs for renoprotective effects on patients with diabetes but interpretation was limited by the surrogate renal outcomes or the male veteran population [18–19]. Our study aimed to compare ACEIs with ARBs for major renal outcomes and survival in a 15-year cohort of patients with diabetes, and assess the effects among patients with different age and comorbid diseases.

## Materials and methods

### Data sources

This cohort study utilized data from the Longitudinal Cohort of Diabetes Patients (LHDB) from the National Health Insurance (NHI) Research Database of Taiwan, which is constructed and maintained by the National Health Research Institutes of Taiwan. The NHI system covers more than 99% of Taiwan’s population and has been in operation since 1995 [20, 21]. The LHDB is a sub-dataset comprising a randomly sampled cohort of de-identified patients with diabetes (http://nhird.nhri.org.tw/en/Data_Subsets.html#S4). The LHDB defined a patient to have diabetes by matching any one of the following criteria: 1) at least one inpatient record with the diagnosis code of diabetes or the prescription of glucose-lowering drugs; 2) at least two outpatient visits with the diagnosis code of diabetes within one year; or 3) one outpatient visit with the diagnosis code of diabetes, and at least one more outpatient visit with prescription of glucose-lowering drugs within one year. The diagnosis code for diabetes should include the ICD-9-CM (International Classification of Diseases-Ninth Revision-Clinical Modification) code 250 or 648.0, or A-code A181 (corresponds to ICD-9-CM 250.x). For the present study we analyzed 831,692 patients during the period of 1997 to 2011. We obtained their claims data including inpatient records, outpatient records, registries for beneficiaries (including scrambled identification number, birthday, sex, coverage period, geographic location, occupation, and income, etc.), and registries for patients with catastrophic illness (co-payments are waived for patients receiving medical treatments related to the registered diseases). The Institutional Review Board of the National Taiwan University Hospital has approved this study and waived the requirement for informed consent, because the database used in this study had only de-identified information, and linkage to other databases was not allowed. The supporting information (S1 Table) provides a detailed list for oral blood pressure-lowering drugs reimbursed by the NHI of Taiwan.

### Study participants

Taiwan’s guidelines have recommended ACEIs or ARBs as the first-line therapy for patients with diabetes or chronic kidney disease, and Taiwan’s NHI has allowed physicians to freely prescribe either an ACEI or an ARB if the patient was indicated to receive a RAS inhibitor [22, 23]. This study enrolled incident users of ACEIs or ARBs in outpatient records between 1997 and 2010. Because previous studies have shown that prescriptions for three months facilitate the maintenance of medications, and the persistence of blood pressure-lowering drugs are more stable after three months of medication initiation, we assessed patients with at least 90 days of continuous use of oral blood pressure-lowering drugs [24–26]. The first date of the prescription of blood pressure-lowering drugs was defined as the index date. We obtained the duration of blood pressure-lowering drug exposure as days of use for each prescription from outpatient records, but not inpatient records because those data were not provided in the original claims data. We defined ‘continuous use’ if the number of discontinued days was less than seven days. Patients below 18 years of age or who received long-term dialysis before the index date were excluded. We also excluded patients with outcomes occurring within 90 days after the index date. Patients who did not receive ACEIs or ARBs at index date (the ACEI/ARB nonusers), and patients who used both ACEIs and ARBs at index date (the ACEI+ARB combination users), would not be enrolled.

### Study design and exposure assessment

We categorized the participants into either (1) ACEI therapy or (2) ARB therapy based on the prescription at the index date. Data including age, sex, income, occupation, and geographic location at the index date were recorded. We defined the participants’ comorbidities by diagnosis codes from inpatient and outpatient records within one year before the index date [27, 28]. The comorbidities included cardiovascular disease (coronary artery disease, congestive heart failure, and peripheral vascular disease), cerebrovascular disease, chronic pulmonary disease, rheumatologic disease, peptic ulcer disease, liver disease, chronic complications of diabetes, hemiplegia, paraplegia, chronic kidney disease, cancer, and acquired immune deficiency syndrome [29]. Charlson comorbidity index (CCI) scores were calculated based on the comorbidities, in order to quantify patient comorbidity profiles [29].

### Primary and secondary outcomes

The primary outcome of this study was long-term dialysis. Secondary outcomes were hospitalization for acute kidney injury, hospitalization for hyperkalemia, all-cause death, cardiovascular death, and non-cardiovascular death. The observation period started from the index date to the date of the outcome or on December 31, 2011, whichever occurred earlier. Because results of laboratory exams were not recorded in the original claims data, we defined the outcomes by specific diagnostic codes in the registries for patients with catastrophic illness and the inpatient records. Patients with end-stage renal disease requiring long-term hemodialysis or peritoneal dialysis would be registered as catastrophic illness patients with the ICD-9-CM code of 585, and the date on which long-term dialysis began was defined by the application date for this registry [21]. The date of hospitalization for acute kidney injury was defined as the admission date of the first hospitalization with ICD-9-CM code of 584 [30]. The date of hospitalization for hyperkalemia was defined as the admission date of the first hospitalization with ICD-9-CM code of 276.7 [7].

As the LHDB does not provide the data on mortality, and the linkage to other administrative databases (including the death registrations) is not allowed, we defined the status and the date of death as the following conditions: 1) the discharge date of hospitalization if the patient’s record indicated that the patient had died in the hospital, or 2) the date of withdrawal from NHI, if the record indicated that the patient had chosen to die at home and therefore left the hospital against medical advice. Because Taiwan’s NHI is a compulsory single-payer program, the only reason for withdrawal under such a condition would be death [31]. Patients with the outcome of all-cause death were further classified into the outcomes of cardiovascular death or non-cardiovascular death. Cardiovascular death was defined by the last hospitalization with diagnosis code related to cardiovascular causes [32, 33], including myocardial infarction (ICD-9-CM code 410), heart failure (428), cerebrovascular accident (430–437), or sudden cardiac death (427.5, 798). Non-cardiovascular death was defined as death due to all other causes [32, 33].

### Statistical analysis

The distributional properties of continuous data are expressed as mean ± standard deviation and the categorical data are presented as frequency with percentage. For the descriptive statistics, univariable analyses were performed using the independent two-sample *t* test, Wilcoxon signed-rank test, Pearson’s chi-squared test, or Spearman's rank correlation coefficient, as appropriate. Kaplan-Meier cumulative incidence plots were constructed to show time to event for each outcome, and the log-rank test was used to compare ACEI users with ARB users. We used univariable and multivariable Cox proportional hazards models to estimate the hazard ratios (HRs) and 95% confidence intervals (CIs) for study outcomes comparing ACEI therapy with ARB therapy. The analysis was based on an intention-to-treat analysis according to the participants’ blood pressure-lowering drug exposure at index date. We adjusted the multivariable models for age, sex, comorbidities, income, occupation, geographic location, CCI score, and the year of index date. The proportional hazards assumption was assessed using the test of weighted Schoenfeld residuals [34].

To examine whether treatment effects of RAS inhibitors varied among participants with different characteristics, we conducted priori subgroup analyses and interaction tests for participants with different age (< 50 years, 50–65 years, and ≥ 65 years), CCI scores (0–3, 4–5, and ≥ 6), and the presence of specific comorbid diseases (cardiovascular disease, chronic kidney disease, liver disease, and cancer). To evaluate the possibility of a cohort effect among different calendar years, we categorized the participants by years of index date (1997–2003, 2004–2007, and 2008–2010) and assessed the influence by subgroup analyses and interaction tests. For assessing the treatment effects among different follow-up time periods, considering the switch and discontinuation of treatments in later follow-up periods, as well as evaluating the failure of some Cox models to meet the proportional hazards assumption, we performed analysis by partitioning the follow-up time into three intervals (0–5 years, 5–10 years, and ≥ 10 years). This study had 80% power to detect a relative risk reduction of 15% in the primary outcome. Two-sided *P* values ≤ 0.05 were considered statistically significant. Statistical analyses were performed with SAS (version 9.2, SAS Institute, Cary, NC, USA) and Stata software (version 11.1, StataCorp LP, College Station, TX, USA).

## Results

**Fig 1:**
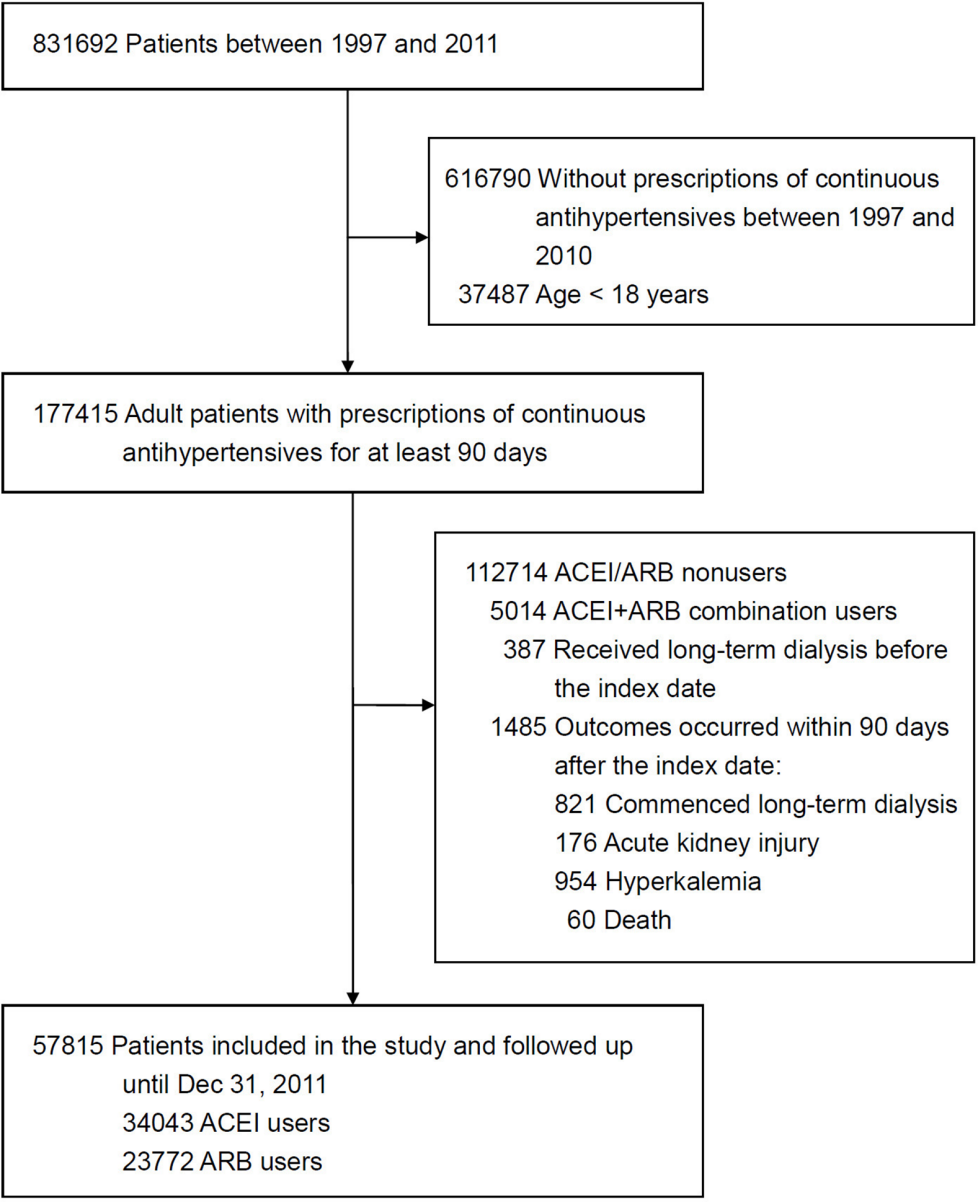
Summary of patient selection. ACEI, angiotensin-converting enzyme inhibitor; ARB, angiotensin II receptor blocker.

The patient selection process is shown in Fig 1. During the study period, 177,415 adult patients received prescription of continuous blood pressure-lowering drugs for at least 90 days. After excluding ACEI/ARB nonusers and ACEI+ARB combination users, as well as those who received long-term dialysis before the index date or those with outcomes occurring within 90 days after the index date, a total of 57,815 patients were enrolled in the study and followed up until December 31, 2011.

### Patient characteristics

Among the participants, 34,043 (58.9%) received ACEI therapy and 23,772 (41.1%) received ARB therapy. The participants had a mean age of 60.1 years and a female prevalence of 43.8%; and they contributed to a total of 457,742 to 461,611 patient-years of follow-up, depending on the outcome analyzed. Participants who received ACEI therapy were older and had a higher prevalence of male sex and cardiovascular disease (Table 1). In addition, the ACEI users had a lower CCI score and a lower prevalence of chronic kidney disease, liver disease, and cancer (Table 1).

**Table 1:** Baseline characteristics of study participants.

	ACEI users	ARB users	
Characteristic	(n = 34043)	(n = 23772)	*P* value
Age, year	60.5 ± 12.9	59.5 ± 12.8	< 0.001
Age group, year			< 0.001
<50	7912 (23.2)	5960 (25.1)	
50–65	12636 (37.1)	9310 (39.2)	
≥65	13495 (39.6)	8502 (35.8)	
Female sex	14350 (42.2)	10977 (46.2)	< 0.001
Comorbidities			
Cardiovascular disease	11500 (33.8)	5285 (22.2)	< 0.001
Chronic kidney disease	4806 (14.1)	4110 (17.3)	< 0.001
Liver disease	7941 (23.3)	7243 (30.5)	< 0.001
Cancer	1558 (4.6)	1818 (7.6)	< 0.001
Charlson comorbidity index			<0.001
0–3	8184 (24.0)	3623 (15.2)	
4–5	13478 (39.6)	9502 (40.0)	
≥6	12381 (36.4)	10647 (44.8)	
Geographic location			<0.001
Northern	12900 (37.9)	11289 (47.5)	
Middle	7733 (22.7)	4780 (20.1)	
Southern	11224 (33.0)	6289 (26.5)	
Eastern or other islands	2186 (6.4)	1414 (5.9)	
Occupation			<0.001
White collar	14668 (43.1)	10939 (46.0)	
Blue collar	14923 (43.8)	10261 (43.2)	
Others	4452 (13.1)	2572 (10.8)	
Income, NTD per month			<0.001
<15000	11129 (32.7)	7002 (29.5)	
15000–30000	18613 (54.7)	13389 (56.3)	
≥30000	4301 (12.6)	3381 (14.2)	
Year of index date			<0.001
1997–2003	15518 (45.6)	3849 (16.2)	
2004–2007	12033 (35.3)	10322 (43.4)	
2008–2010	6492 (19.1)	9601 (40.4)	

Data are expressed as mean ± standard deviation for continuous variables and number (percentage) for categorical variables.

ACEI, angiotensin-converting enzyme inhibitor; ARB, angiotensin II receptor blocker; NTD, New Taiwan Dollar.

#### Outcomes comparing ACEI therapy with ARB therapy in the main analyses

**Fig 2:**
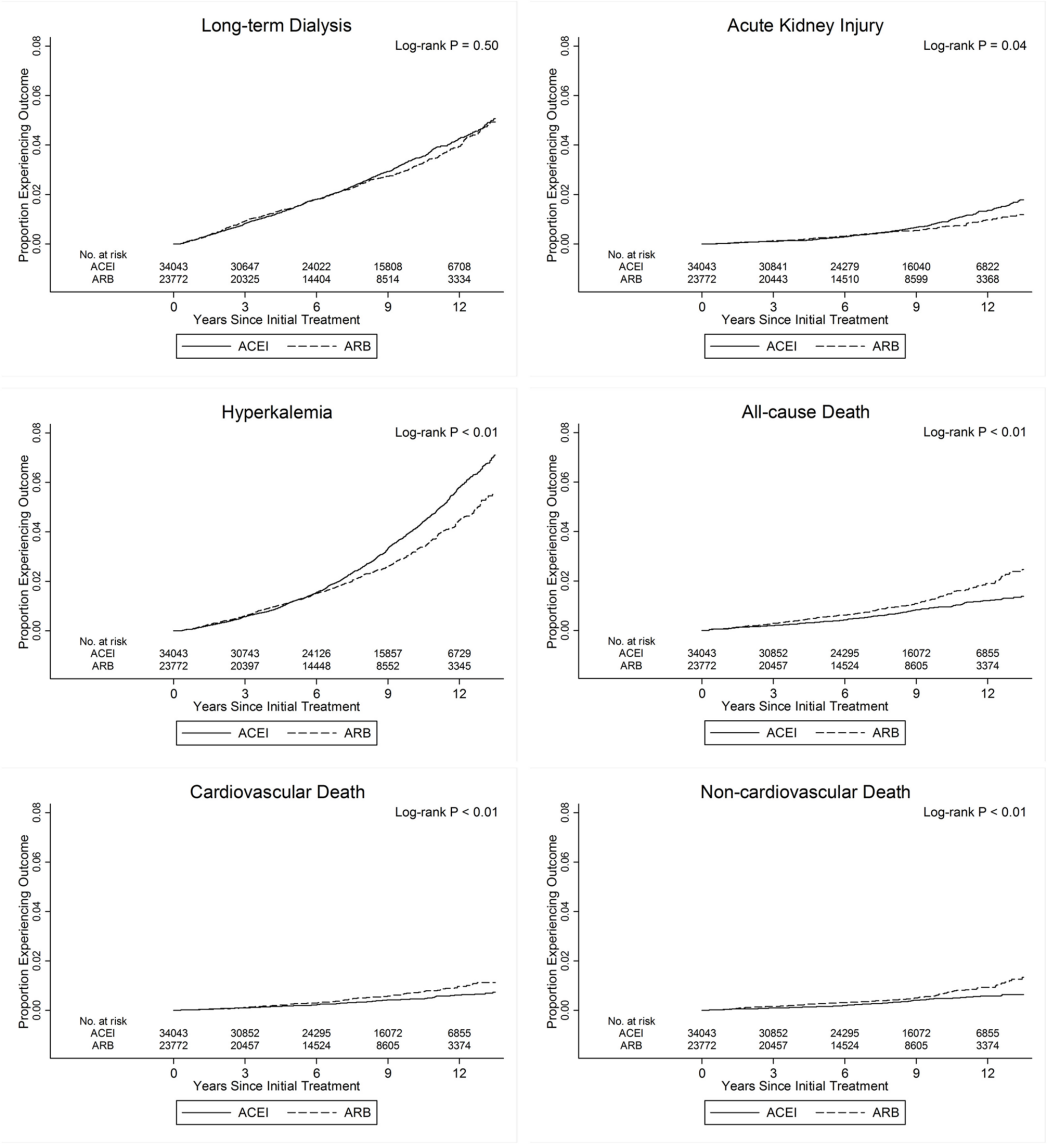
Kaplan-Meier cumulative incidence of time to event for long-term dialysis, acute kidney injury, hyperkalemia, all-cause death, cardiovascular death, and non-cardiovascular death in patients treated with ACEI or ARB. ACEI, angiotensin-converting enzyme inhibitor; ARB, angiotensin II receptor blocker.

Table 2 lists the number of events, incidence rates, and the results of Cox proportional hazards models for each study outcome in the main analyses. There were 1,548 long-term dialysis, 393 hospitalizations of acute kidney injury, 1,751 hospitalizations of hyperkalemia, 506 all-cause deaths, 256 cardiovascular deaths, and 250 non-cardiovascular deaths among the 57,815 study participants. Kaplan-Meier cumulative incidence plots showed no differences in the risk for long-term dialysis between ACEI therapy and ARB therapy (Fig 2). The plots showed increased risks of hospitalization for acute kidney injury and hyperkalemia, but a decreased risk of all-cause death, cardiovascular death, and non-cardiovascular death among the ACEI users. For the primary and secondary outcomes, the adjusted HRs showed no difference between ACEI therapy and ARB therapy. The proportional hazards assumption was met for the outcomes of long-term dialysis, all-cause death, cardiovascular death, and non-cardiovascular death, but not for hospitalization of acute kidney injury and hyperkalemia. After partitioning follow-up time into three periods and repeated the analyses to evaluate the nature of nonproportionality, the proportional hazards assumption was met for each follow-up interval among all outcomes (Table 3).

**Table 2:** Incidence rates and hazard ratios for study outcomes comparing ACEI therapy with ARB therapy.

	Events, No.		Incidence rate per 1000 patient-years		Hazard ratio (95% confidence interval)	
Outcome	ACEI	ARB	ACEI	ARB	Crude	Fully adjusted*
Long-term dialysis	975	573	3.45	3.26	1.04 (0.93–1.15)	0.93 (0.83–1.03)
Acute kidney injury	269	124	0.95	0.70	1.25 (1.01–1.55)†	1.07 (0.85–1.35)
Hyperkalemia	1183	568	4.18	3.23	1.22 (1.11–1.35)†	1.02 (0.92–1.14)
All-cause death	263	243	0.92	1.38	0.65 (0.54–0.77)†	1.17 (0.98–1.40)
Cardiovascular death	136	120	0.48	0.68	0.68 (0.53–0.87)†	1.04 (0.80–1.34)
Non-cardiovascular death	127	123	0.45	0.70	0.62 (0.48–0.79)†	1.28 (0.99–1.65)

* Cox proportional hazards model adjusted for age, sex, cardiovascular disease, chronic kidney disease, hepatic disease, cancer, income, occupation, geographic location, Charlson comorbidity index score, and year of index date.

† *P* ≤ 0.05.

ACEI, angiotensin-converting enzyme inhibitor; ARB, angiotensin II receptor blocker.

**Table 3:** Hazard ratios (95% confidence interval)* for study outcomes comparing ACEI therapy with ARB therapy, by partitioning the follow-up time.

	Follow-up time		
Outcome	0–5 years	5–10 years	> 10 years
Long-term dialysis	1.01 (0.87–1.18)	0.89 (0.74–1.07)	0.70 (0.51–0.95) †
Acute kidney injury	0.82 (0.57–1.20)	1.10 (0.76–1.58)	1.51 (0.90–2.51)
Hyperkalemia	0.96 (0.81–1.14)	1.07 (0.90–1.26)	1.01 (0.78–1.29)
All-cause death	0.99 (0.75–1.30)	1.48 (1.11–1.97)†	1.06 (0.67–1.68)
Cardiovascular death	0.94 (0.64–1.38)	1.05 (0.70–1.56)	1.33 (0.69–2.54)
Non-cardiovascular death	1.01 (0.69–1.49)	2.00 (1.32–3.03)†	0.75 (0.38–1.48)

* Cox proportional hazards model adjusted for age, sex, cardiovascular disease, chronic kidney disease, hepatic disease, cancer, income, occupation, geographic location, Charlson comorbidity index score, and year of index date.

†*P* ≤ 0.05.

ACEI, angiotensin-converting enzyme inhibitor; ARB, angiotensin II receptor blocker.

### Subgroup analyses and tests for interaction

**Fig 3:**
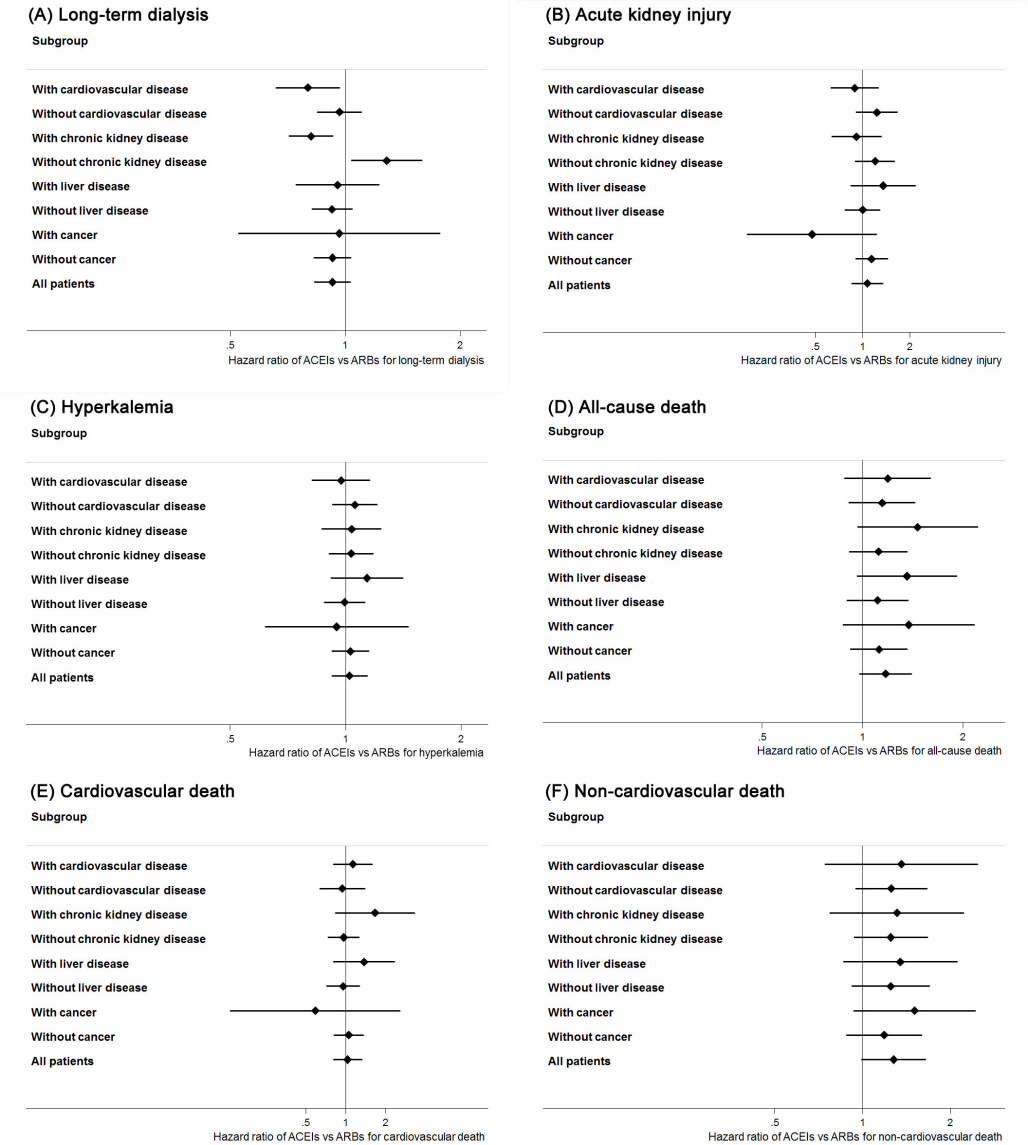
**Hazard ratios and 95% confidence intervals comparing ACEI therapy with ARB therapy for study outcomes of (A) Long-term dialysis; (B) Acute kidney injury; (C) Hyperkalemia; (D) All-cause death; (E) Cardiovascular death; and (F) Non-cardiovascular death.** ACEI, angiotensin-converting enzyme inhibitor; ARB, angiotensin II receptor blocker.

Table 4, Table 5 and Fig 3 show the results of the subgroup analyses. Compared with ARB therapy, ACEI therapy showed significantly lower hazard for long-term dialysis among participants with cardiovascular disease (HR 0.80, 95% CI 0.66–0.97) or chronic kidney disease (0.81, 0.71–0.93), and the interaction tests were also significant (*P* = 0.003 for cardiovascular disease; *P* = 0.001 for chronic kidney disease). In addition, ARB therapy demonstrated a lower hazard for long-term dialysis among participants without chronic kidney disease. ACEI therapy showed a lower hazard for long-term dialysis among the participants of the highest category of CCI scores, but the interaction test was not significant (HR 0.83, 95% CI 0.72–0.96, interaction *P* = 0.09). Analyses in the subgroups of different age, year of index date, as well as those with liver disease or cancer, showed similar results as the main analysis for long-term dialysis. Most of the subgroup analyses for secondary outcomes showed non-significant results, except the lower hazards in ACEI therapy for cardiovascular death among participants with index dates during the period of 1997 to 2003 (interaction *P* = 0.007), and the lower hazards in ARB therapy for acute kidney injury among participants with index dates during the period of 2004 to 2007 (interaction *P* = 0.04) or all-cause death among participants with CCI scores of 4–5 (interaction *P* = 0.04). ARB therapy revealed a borderline lower hazard for non-cardiovascular death among participants with CCI scores ≥ 6 but the interaction test was not significant (interaction *P* = 0.19).

**Table 4:** Hazard ratios (95% confidence interval)* for study outcomes comparing ACEI therapy with ARB therapy, by subgroups of specific comorbid disease.

	**With cardiovascular disease**	**Without cardiovascular disease**
**Outcome**	**(n = 16785)**	**(n = 41030)**
Long-term dialysis	0.80 (0.66–0.97)†	0.97 (0.84–1.11)
Acute kidney injury	0.89 (0.62–1.26)	1.23 (0.91–1.67)
Hyperkalemia	0.97 (0.82–1.16)	1.06 (0.92–1.21)
All-cause death	1.19 (0.88–1.60)	1.14 (0.91–1.44)
Cardiovascular death	1.13 (0.80–1.59)	0.94 (0.64–1.40)
Non-cardiovascular death	1.36 (0.74–2.49)	1.26 (0.95–1.67)
	**With chronic kidney disease**	**Without chronic kidney disease**
**Outcome**	**(n = 8916)**	**(n = 48899)**
Long-term dialysis	0.81 (0.71–0.93)†	1.28 (1.04–1.59)†
Acute kidney injury	0.91 (0.63–1.32)	1.20 (0.89–1.61)
Hyperkalemia	1.04 (0.87–1.24)	1.03 (0.90–1.18)
All-cause death	1.46 (0.96–2.22)	1.11 (0.91–1.36)
Cardiovascular death	1.66 (0.83–3.31)	0.97 (0.73–1.27)
Non-cardiovascular death	1.31 (0.77–2.23)	1.25 (0.93–1.67)
	**With liver disease**	**Without liver disease**
**Outcome**	**(n = 15184)**	**(n = 42631)**
Long-term dialysis	0.95 (0.74–1.23)	0.92 (0.82–1.04)
Acute kidney injury	1.35 (0.84–2.18)	1.00 (0.77–1.30)
Hyperkalemia	1.14 (0.91–1.41)	0.99 (0.88–1.12)
All-cause death	1.36 (0.96–1.92)	1.11 (0.90–1.37)
Cardiovascular death	1.38 (0.80–2.35)	0.96 (0.72–1.28)
Non-cardiovascular death	1.35 (0.86–2.12)	1.25 (0.92–1.70)
	**With cancer**	**Without cancer**
**Outcome**	**(n = 3376)**	**(n = 54439)**
Long-term dialysis	0.96 (0.52–1.77)	0.93 (0.83–1.04)
Acute kidney injury	0.48 (0.18–1.23)	1.14 (0.90–1.45)
Hyperkalemia	0.95 (0.62–1.46)	1.03 (0.92–1.15)
All-cause death	1.37 (0.87–2.16)	1.12 (0.92–1.36)
Cardiovascular death	0.59 (0.14–2.58)	1.06 (0.81–1.37)
Non-cardiovascular death	1.51 (0.93–2.44)	1.19 (0.88–1.60)

* Cox proportional hazards model adjusted for age, sex, cardiovascular disease, chronic kidney disease, hepatic disease, cancer, income, occupation, geographic location, Charlson comorbidity index score, and year of index date, except for the covariate which the subgroup was based on.

†*P* ≤ 0.05.

ACEI, angiotensin-converting enzyme inhibitor; ARB, angiotensin II receptor blocker.

**Table 5:** Hazard ratios (95% confidence interval)* for study outcomes comparing ACEI therapy with ARB therapy, by subgroups of different age, Charlson comorbidity index score, or year of index date.

	**Age**		
	**< 50 years**	**50–65 years**	**≥ 65 years**
**Outcome**	**(n = 13872)**	**(n = 21946)**	**(n = 21997)**
Long-term dialysis	0.97 (0.78–1.20)	0.94 (0.78–1.13)	0.90 (0.75–1.08)
Acute kidney injury	0.85 (0.38–1.88)	0.65 (0.41–1.05)	1.31 (0.99–1.74)
Hyperkalemia	0.83 (0.60–1.13)	0.89 (0.71–1.11)	1.13 (0.99–1.29)
All-cause death	1.32 (0.82–2.11)	1.19 (0.85–1.66)	1.14 (0.89–1.45)
Cardiovascular death	1.06 (0.56–2.01)	1.37 (0.86–2.18)	0.90 (0.63–1.28)
Non-cardiovascular death	1.72 (0.85–3.48)	1.00 (0.62–1.62)	1.39 (0.99–1.95)
	**Charlson comorbidity index score**		
	**0–3**	**4–5**	**≥ 6**
**Outcome**	**(n = 11807)**	**(n = 22980)**	**(n = 23028)**
Long-term dialysis	1.31 (0.90–1.90)	1.04 (0.84–1.28)	0.83 (0.72–0.96)†
Acute kidney injury	1.26 (0.62–2.56)	1.17 (0.77–1.79)	1.02 (0.76–1.38)
Hyperkalemia	1.21 (0.86–1.71)	1.04 (0.86–1.25)	1.00 (0.86–1.15)
All-cause death	0.72 (0.43–1.21)	1.38 (1.01–1.86)†	1.21 (0.94–1.56)
Cardiovascular death	0.53 (0.26–1.11)	1.45 (0.93–2.26)	1.00 (0.71–1.43)
Non-cardiovascular death	0.98 (0.48–2.02)	1.28 (0.84–1.96)	1.44 (1.00–2.07)†
	**Year of index date**		
	**1997–2003**	**2004–2007**	**2008–2010**
**Outcome**	**(n = 19367)**	**(n = 22355)**	**(n = 16093)**
Long-term dialysis	0.94 (0.78–1.12)	0.92 (0.78–1.08)	0.86 (0.63–1.18)
Acute kidney injury	0.86 (0.61–1.22)	1.44 (1.02–2.04)†	0.70 (0.37–1.32)
Hyperkalemia	0.95 (0.80–1.12)	1.12 (0.95–1.32)	0.87 (0.65–1.18)
All-cause death	0.46 (0.16–1.26)	1.27 (0.97–1.68)	1.16 (0.90–1.48)
Cardiovascular death	0.27 (0.08–0.92)†	1.12 (0.75–1.65)	1.07 (0.76–1.52)
Non-cardiovascular death	1.44 (0.16–12.9)	1.38 (0.94–2.03)	1.20 (0.84–1.69)

* Cox proportional hazards model adjusted for age, sex, cardiovascular disease, chronic kidney disease, hepatic disease, cancer, income, occupation, geographic location, Charlson comorbidity index score, and year of index date, except for the covariate which the subgroup was based on.

†*P* ≤ 0.05.

ACEI, angiotensin-converting enzyme inhibitor; ARB, angiotensin II receptor blocker.

## Discussion

In this cohort study of patients with diabetes, the main analyses showed no difference in the outcomes of long-term dialysis, hospitalization for acute kidney injury, hospitalization for hyperkalemia, all-cause death, cardiovascular death, and non-cardiovascular death between ACEI therapy and ARB therapy. However, subgroup analyses and interaction tests suggested that ACEI therapy might provide additional protective effect against long-term dialysis in patients with cardiovascular disease and chronic kidney disease.

### Strengths of this study

The strength of this study is its large sample size in a 15-year nationally representative population with diabetes, which is possible to provide sufficient statistical power in major renal outcomes. We applied rigorous methods for survival analyses, including multivariate adjustment of potential confounding factors, subgroup analyses, and interaction tests for important covariates. We also evaluated the assumption of proportional hazard, and partitioned the follow-up time to assess the treatment effects among different time periods. The analyses during the second and the third interval were similar to the main analyses, which showed the consistency and robustness for treatment effects during long-term follow-up periods. In addition, the primary outcome was highly accurate because the registration as a catastrophic-illness patient needing long-term dialysis must meet strict criteria and be submitted by a nephrologist, and that need must be verified by at least two other senior nephrologists [21].

### Results in relation to other studies and reviews

Only a few randomized clinical trials comparing ACEI therapy with ARB therapy were powered to evaluate renal outcomes in patients with diabetes. The Diabetics Exposed to Telmisartan and Enalapril study, which evaluated 250 patients with type 2 diabetes, reported that enalapril was not significantly superior to telmisartan for the five-year reduction of glomerular filtration rate (14.9 ml/min/1.73 m^2^ reduction compared with 17.9 ml/min/1.73 m^2^ reduction), and also showed non-significant difference in the change of serum creatinine and urinary albumin excretion [35]. The Renin-Angiotensin System Study [36], which evaluated 94 enalapril and 96 losartan users with type 1 diabetes for five years, reported that enalapril users had a non-significantly lower change in glomerular mesangial fraction volume (0.005 ± 0.050 units) and urinary albumin excretion rate (7.7±15.5 μg/min) compared with losartan users (0.026 ± 0.054 units; 10.6 ± 17.6 μg/min). However, those studies did not report major renal outcomes such as long-term dialysis or hospitalization for acute kidney injury, and their sample size were relatively limited.

In a cohort study of 16,489 patients with type 2 diabetes and normoalbuminuria, Al-Sayed showed that ACEI users had lower risk of albuminuria than ARB users (HR 0.77, 95% CI 0.62–0.95), but they did not evaluate long-term renal outcomes [19]. In another cohort study of 5,166 patients with diabetic kidney disease, Campbell reported a better protective effect comparing ACEIs with ARBs for the outcome of long-term dialysis (odds ratio 0.33, 95% CI 0.13–0.82), but the study was limited to the male veterans population [18]. Network meta-analysis estimates the interrelations across multiple treatments comparisons and provides the ranking of each treatment in randomized clinical trials, studies by us and Palmer both revealed a non-significant difference between ACEIs and ARBs in patients with diabetes, but ACEIs consistently showed higher probabilities of being protective in the superior ranking positions for renal outcomes such as long-term dialysis, acute kidney injury, or doubling of creatinine [6, 37]. These findings imply that small differences might exist between ACEIs and ARBs for patients with diabetes, and ACEIs might provide additional renoprotective effects through the elevation in bradykinin as well as the activation of B2-type bradykinin receptors [38, 39]. In the present study, why ACEI and ARB users showed differences in subgroups with specific comorbidities could partially be explained by the median follow-up time of eight years, a duration that is probably inadequate to detect differences for long-term dialysis in the entire study population but detectable in patients with specific comorbid diseases, such as cardiovascular disease or chronic kidney disease. The lower hazard of ARB therapy in patients without chronic kidney disease might be related to the shorter follow-up time among ARB users. Because ARBs belong to a newer class of RAS inhibitors, and more patients received ACEIs during the earlier time periods.

### Study limitations

Our study has several limitations. First, this cohort was based on claims data, and did not contain covariates of laboratory exams, blood pressure, body mass index, lifestyle behavior, and prescription compliance. Therefore, it was not possible to classify participants or adjust models according to the stages of chronic kidney disease, levels of blood pressure, or adequacy of metabolic control. As these covariates would influence outcomes and were commonly associated with comorbid diseases [4, 5, 40], we adjusted comorbidities in the models and analyzed comorbidities in subgroup analyses in order to minimize influences from these unmeasured covariates. Second, this study was based on an intention-to-treat analysis design and participants might have switched or discontinued their treatments during later follow-up periods. To minimize the influences of medication adherence and persistence, we assessed the effects under different partitioned periods, which showed similar results for the primary and secondary outcomes in long-term follow-up periods. Third, unmeasured confounders were unavoidable owing to the observational nature of this cohort study. Some of the baseline characteristics were different between ACEI users and ARB users, such as age, sex, comorbidities, and socioeconomic status, etc. While Taiwan’s NHI has allowed physicians to freely prescribe either an ACEI or an ARB for patients with diabetes, indication bias such as physician preference or patient intolerance could still exist after adjusting for all available covariates in the Cox proportional hazard models. Besides, the follow-up time of this study may not be long enough to detect differences of effects for the whole study population. A randomized clinical trial powered for long-term major renal outcomes in patients with diabetes can possibly make the conclusion, yet conducting such a trial can be challenging, and our study provides valuable information for future trial design. Finally, given that this is a cohort of patients with diabetes from a country mainly consists of Asian ethnic groups, external generalization of our findings to population without diabetes or other ethnic groups requires additional studies.

### Conclusion

Our analyses show similar effects of ACEIs and ARBs in patients with diabetes. However, ACEIs might provide additional renoprotective effects among patients with diabetes who have cardiovascular disease or chronic kidney disease.

## Supporting information

S1 File STROBE checklist. (DOCX)

S1 Table Oral blood pressure-lowering drugs reimbursed by the National Health Insurance of Taiwan. (DOCX)

## References

[1] Tsai WC, Wu HY, Peng YS, Yang JY, Chen HY, Chiu YL, et al Association of Intensive Blood Pressure Control and Kidney Disease Progression in Nondiabetic Patients With Chronic Kidney Disease: A Systematic Review and Meta-analysis [published online Mar 13, 2017]. JAMA Intern Med. 10.1001/jamainternmed.2017.0197 PMC5818822 28288249

[2] Tsai WC, Wu HY, Peng YS, Ko MJ, Wu MS, Hung KY, et al Risk Factors for Development and Progression of Chronic Kidney Disease: A Systematic Review and Exploratory Meta-Analysis. Medicine (Baltimore). 2016;95(11):e3013. 26986114 10.1097/MD.0000000000003013 PMC4839895

[3] Wu HY, Peng YS, Chiang CK, Huang JW, Hung KY, Wu KD, et al Diagnostic performance of random urine samples using albumin concentration vs ratio of albumin to creatinine for microalbuminuria screening in patients with diabetes mellitus: a systematic review and meta-analysis. JAMA Intern Med. 2014;174(7):1108–15. doi: 10.1001/jamainternmed.2014.1363 24798807 10.1001/jamainternmed.2014.1363

[4] Jha V, Garcia-Garcia G, Iseki K, Li Z, Naicker S, Plattner B, et al Chronic kidney disease: global dimension and perspectives. Lancet. 2013;382(9888):260–72. doi: 10.1016/S0140-6736(13)60687-X 23727169 10.1016/S0140-6736(13)60687-X

[5] Fukuma S, Shimizu S, Niihata K, Sada KE, Yanagita M, Hatta T, et al Development of quality indicators for care of chronic kidney disease in the primary care setting using electronic health data: a RAND-modified Delphi method [published online May 4, 2016]. Clin Exp Nephrol. 10.1007/s10157-016-1274-8 27145768

[6] Wu HY, Huang JW, Lin HJ, Liao WC, Peng YS, Hung KY, et al Comparative effectiveness of renin-angiotensin system blockers and other antihypertensive drugs in patients with diabetes: systematic review and bayesian network meta-analysis. BMJ. 2013;347:f6008 doi: 10.1136/bmj.f6008 24157497 10.1136/bmj.f6008 PMC3807847

[7] Hsu TW, Liu JS, Hung SC, Kuo KL, Chang YK, Chen YC, et al Renoprotective effect of renin-angiotensin-aldosterone system blockade in patients with predialysis advanced chronic kidney disease, hypertension, and anemia. JAMA Intern Med. 2014;174(3):347–54. doi: 10.1001/jamainternmed.2013.12700 24343093 10.1001/jamainternmed.2013.12700

[8] Iseki K, Arima H, Kohagura K, Komiya I, Ueda S, Tokuyama K, et al Effects of angiotensin receptor blockade (ARB) on mortality and cardiovascular outcomes in patients with long-term haemodialysis: a randomized controlled trial. Nephrol Dial Transplant. 2013;28(6):1579–89. doi: 10.1093/ndt/gfs590 23355629 10.1093/ndt/gfs590

[9] Hayashi M, Uchida S, Kawamura T, Kuwahara M, Nangaku M, Iino Y. Prospective randomized study of the tolerability and efficacy of combination therapy for hypertensive chronic kidney disease: results of the PROTECT-CKD study. Clin Exp Nephrol. 2015;19(5):925–32. doi: 10.1007/s10157-015-1091-5 25680887 10.1007/s10157-015-1091-5

[10] Mancia G, Fagard R, Narkiewicz K, Redon J, Zanchetti A, Bohm M, et al 2013 ESH/ESC guidelines for the management of arterial hypertension: the Task Force for the Management of Arterial Hypertension of the European Society of Hypertension (ESH) and of the European Society of Cardiology (ESC). Eur Heart J. 2013;34(28):2159–219. doi: 10.1093/eurheartj/eht151 23771844 10.1093/eurheartj/eht151

[11] James PA, Oparil S, Carter BL, Cushman WC, Dennison-Himmelfarb C, Handler J, et al 2014 evidence-based guideline for the management of high blood pressure in adults: report from the panel members appointed to the Eighth Joint National Committee (JNC 8). JAMA. 2014;311(5):507–20. doi: 10.1001/jama.2013.284427 24352797 10.1001/jama.2013.284427

[12] Kidney Disease: Improving Global Outcomes (KDIGO) CKD Work Group. KDIGO 2012 Clinical Practice Guideline for the Evaluation and Management of Chronic Kidney Disease. Kidney Int Suppl. 2013;3:1–150.

[13] American Diabetes Association. Cardiovascular disease and risk management. Sec. 9. In Standards of Medical Care in Diabetes—2017. Diabetes Care. 2017;40 Suppl 1:S75–87. doi: 10.2337/dc17-S012 27979896

[14] Pawluczyk IZ, Patel SR, Harris KP. The role of bradykinin in the antifibrotic actions of perindoprilat on human mesangial cells. Kidney Int. 2004;65(4):1240–51. doi: 10.1111/j.1523-1755.2004.00494.x 15086463 10.1111/j.1523-1755.2004.00494.x

[15] Mann JF, Schmieder RE, McQueen M, Dyal L, Schumacher H, Pogue J, et al Renal outcomes with telmisartan, ramipril, or both, in people at high vascular risk (the ONTARGET study): a multicentre, randomised, double-blind, controlled trial. Lancet. 2008;372(9638):547–53. doi: 10.1016/S0140-6736(08)61236-2 18707986 10.1016/S0140-6736(08)61236-2

[16] Tobe SW, Clase CM, Gao P, McQueen M, Grosshennig A, Wang X, et al Cardiovascular and renal outcomes with telmisartan, ramipril, or both in people at high renal risk: results from the ONTARGET and TRANSCEND studies. Circulation. 2011;123(10):1098–107. doi: 10.1161/CIRCULATIONAHA.110.964171 21357827 10.1161/CIRCULATIONAHA.110.964171

[17] Wu HY, Hung KY, Tu YK, Chien KL. Comparative effectiveness of antihypertensive drugs in patients with diabetes. J Comp Eff Res. 2014;3(3):213–5. doi: 10.2217/cer.14.10 24969144 10.2217/cer.14.10

[18] Campbell HM, Khan N, Raisch DW, Borrego ME, Sather MR, Murata GH. Angiotensin-converting enzyme inhibitors versus angiotensin receptor blockers for end-stage renal disease/mortality in type 2 diabetes. Diabetes Res Clin Pract. 2013;102(3):233–41. doi: 10.1016/j.diabres.2013.10.005 24183258 10.1016/j.diabres.2013.10.005

[19] Al-Sayed NA, Gao T, Wells BJ, Yu C, Zimmerman RS. Angiotensin-converting enzyme inhibitors reduce albuminuria more than angiotensin receptor blockers in patients with type 2 diabetes. Endocr Pract. 2013;19(4):579–86. doi: 10.4158/EP12272.OR 23425642 10.4158/EP12272.OR

[20] Hsing AW, Ioannidis JP. Nationwide Population Science: Lessons From the Taiwan National Health Insurance Research Database. JAMA Intern Med. 2015;175(9):1527–9. doi: 10.1001/jamainternmed.2015.3540 26192815 10.1001/jamainternmed.2015.3540

[21] Yang DC, Lee LJ, Hsu CC, Chang YY, Wang MC, Lin WH, et al Estimation of expected life-years saved from successful prevention of end-stage renal disease in elderly patients with diabetes: a nationwide study from Taiwan. Diabetes Care. 2012;35(11):2279–85. doi: 10.2337/dc12-0545 22875232 10.2337/dc12-0545 PMC3476928

[22] Chiang CE, Wang TD, Li YH, Lin TH, Chien KL, Yeh HI, et al 2010 guidelines of the Taiwan Society of Cardiology for the management of hypertension. J Formos Med Assoc. 2010;109(10):740–73. doi: 10.1016/S0929-6646(10)60120-9 20970072 10.1016/S0929-6646(10)60120-9

[23] Chiang CE, Wang TD, Ueng KC, Lin TH, Yeh HI, Chen CY, et al 2015 guidelines of the Taiwan Society of Cardiology and the Taiwan Hypertension Society for the management of hypertension. J Chin Med Assoc. 2015;78(1):1–47. doi: 10.1016/j.jcma.2014.11.005 25547819 10.1016/j.jcma.2014.11.005

[24] Patel BV, Remigio-Baker RA, Mehta D, Thiebaud P, Frech-Tamas F, Preblick R. Effects of initial antihypertensive drug class on patient persistence and compliance in a usual-care setting in the United States. J Clin Hypertens (Greenwich). 2007;9(9):692–700. 17786070 10.1111/j.1524-6175.2007.07194.x PMC8109994

[25] Van Wijk BL, Klungel OH, Heerdink ER, de Boer A. Refill persistence with chronic medication assessed from a pharmacy database was influenced by method of calculation. J Clin Epidemiol. 2006;59(1):11–7. doi: 10.1016/j.jclinepi.2005.05.005 16360556 10.1016/j.jclinepi.2005.05.005

[26] Steiner JF, Robbins LJ, Roth SC, Hammond WS. The effect of prescription size on acquisition of maintenance medications. J Gen Intern Med. 1993;8(6):306–10. 8320574 10.1007/BF02600143

[27] Chen YC, Su YC, Li CY, Wu CP, Lee MS. A nationwide cohort study suggests chronic hepatitis B virus infection increases the risk of end-stage renal disease among patients in Taiwan. Kidney Int. 2015;87(5):1030–8. doi: 10.1038/ki.2014.363 25426815 10.1038/ki.2014.363

[28] Hughes DR, Jiang M, Duszak R Jr. A comparison of diagnostic imaging ordering patterns between advanced practice clinicians and primary care physicians following office-based evaluation and management visits. JAMA Intern Med. 2015;175(1):101–7. doi: 10.1001/jamainternmed.2014.6349 25419763 10.1001/jamainternmed.2014.6349

[29] Deyo RA, Cherkin DC, Ciol MA. Adapting a clinical comorbidity index for use with ICD-9-CM administrative databases. J Clin Epidemiol. 1992;45(6):613–9. 1607900 10.1016/0895-4356(92)90133-8

[30] Chang JW, Jeng MJ, Yang LY, Chen TJ, Chiang SC, Soong WJ, et al The epidemiology and prognostic factors of mortality in critically ill children with acute kidney injury in Taiwan. Kidney Int. 2015;87(3):632–9. doi: 10.1038/ki.2014.299 25252027 10.1038/ki.2014.299

[31] Tung YC, Chang GM. The effect of cuts in reimbursement on stroke outcome: a nationwide population-based study during the period 1998 to 2007. Stroke. 2010;41(3):504–9. doi: 10.1161/STROKEAHA.109.568956 20075356 10.1161/STROKEAHA.109.568956

[32] Hicks KA, Tcheng JE, Bozkurt B, Chaitman BR, Cutlip DE, Farb A, et al 2014 ACC/AHA Key Data Elements and Definitions for Cardiovascular Endpoint Events in Clinical Trials: A Report of the American College of Cardiology/American Heart Association Task Force on Clinical Data Standards (Writing Committee to Develop Cardiovascular Endpoints Data Standards). J Nucl Cardiol. 2015;22(5):1041–144. doi: 10.1007/s12350-015-0209-1 26204990 10.1007/s12350-015-0209-1

[33] Carrero JJ, de Jager DJ, Verduijn M, Ravani P, De Meester J, Heaf JG, et al Cardiovascular and noncardiovascular mortality among men and women starting dialysis. Clin J Am Soc Nephrol. 2011;6(7):1722–30. doi: 10.2215/CJN.11331210 21734088 10.2215/CJN.11331210

[34] Grambsch PM, Therneau TM. Proportional hazards tests and diagnostics based on weighted residuals. Biometrika. 1994;81(3):515–26.

[35] Barnett AH, Bain SC, Bouter P, Karlberg B, Madsbad S, Jervell J, et al Angiotensin-receptor blockade versus converting-enzyme inhibition in type 2 diabetes and nephropathy. N Engl J Med. 2004;351(19):1952–61. doi: 10.1056/NEJMoa042274 15516696 10.1056/NEJMoa042274

[36] Mauer M, Zinman B, Gardiner R, Suissa S, Sinaiko A, Strand T, et al Renal and retinal effects of enalapril and losartan in type 1 diabetes. N Engl J Med. 2009;361(1):40–51. doi: 10.1056/NEJMoa0808400 19571282 10.1056/NEJMoa0808400 PMC2978030

[37] Palmer SC, Mavridis D, Navarese E, Craig JC, Tonelli M, Salanti G, et al Comparative efficacy and safety of blood pressure-lowering agents in adults with diabetes and kidney disease: a network meta-analysis. Lancet. 2015;385(9982):2047–56. doi: 10.1016/S0140-6736(14)62459-4 26009228 10.1016/S0140-6736(14)62459-4

[38] Izzo JL Jr., Weir MR. Angiotensin-converting enzyme inhibitors. J Clin Hypertens (Greenwich). 2011;13(9):667–75. 21896148 10.1111/j.1751-7176.2011.00508.x PMC8108813

[39] Seccia TM, Belloni AS, Guidolin D, Sticchi D, Nussdorfer GG, Pessina AC, et al The renal antifibrotic effects of angiotensin-converting enzyme inhibition involve bradykinin B2 receptor activation in angiotensin II-dependent hypertension. J Hypertens. 2006;24(7):1419–27. doi: 10.1097/01.hjh.0000234124.94013.ac 16794493 10.1097/01.hjh.0000234124.94013.ac

[40] Tozawa M, Iseki K, Iseki C, Kinjo K, Ikemiya Y, Takishita S. Blood pressure predicts risk of developing end-stage renal disease in men and women. Hypertension. 2003;41(6):1341–5. doi: 10.1161/01.HYP.0000069699.92349.8C 12707291 10.1161/01.HYP.0000069699.92349.8C

