# Supplementary material for: Comparative effectiveness of angiotensin-converting enzyme inhibitors versus angiotensin II receptor blockers for major renal outcomes in patients with diabetes: A 15-year cohort study
Source: PLoS One. 2017 May 15;12(5):e0177654. doi: 10.1371/journal.pone.0177654 (PMC5432180; doi:10.1371/journal.pone.0177654)
Supplement: S1 Table — (DOCX) [file pone.0177654.s002.docx]

**S1 Table. Oral blood pressure-lowering drugs reimbursed by the National Health Insurance of Taiwan**

|  | **Angiotensin-converting enzyme inhibitor** | | | |
| --- | --- | --- | --- | --- |
|  | **ATC code** |  | **Generic name** |  |
|  | C09AA07 |  | benazepril |  |
|  | C09AA01 |  | captopril |  |
|  | C09AA08 |  | cilazapril |  |
|  | C09AA02 |  | enalapril |  |
|  | C09AA09 |  | fosinopril |  |
|  | C09AA16 |  | imidapril |  |
|  | C09AA03 |  | lisinopril |  |
|  | C09AA04 |  | perindopril |  |
|  | C09AA06 |  | quinapril |  |
|  | C09AA05 |  | ramipril |  |
|  |  |  |  |  |
|  | **Angiotensin II receptor blocker** | | |  |
|  | **ATC code** |  | **Generic name** |  |
|  | C09CA06 |  | candesartan |  |
|  | C09CA02 |  | eprosartan |  |
|  | C09CA04 |  | irbesartan |  |
|  | C09CA01 |  | losartan |  |
|  | C09CA08 |  | olmesartan medoxomil | |
|  | C09CA07 |  | telmisartan |  |
|  | C09CA03 |  | valsartan |  |
|  |  |  |  |  |
|  | **Calcium channel blocker (dihydropyridine)** | | | |
|  | **ATC code** |  | **Generic name** |  |
|  | C08CA01 |  | amlodipine |  |
|  | C08CA12 |  | barnidipine |  |
|  | C08CA15 |  | benidipine |  |
|  | C08CA02 |  | felodipine |  |
|  | C08CA03 |  | isradipine |  |
|  | C08CA09 |  | lacidipine |  |
|  | C08CA13 |  | lercanidipine |  |
|  | C08CA04 |  | nicardipine |  |
|  | C08CA05 |  | nifedipine |  |
|  | C08CA06 |  | nimodipine |  |
|  | C08CA08 |  | nitrendipine |  |
|  |  |  | **(To be continued)** |  |
| **(Continued)** | | |  |  |
|  | **Calcium channel blocker (non-dihydropyridine)** | | | |
|  | **ATC code** |  | **Generic name** |  |
|  | C08DB01 |  | diltiazem |  |
|  | C08DA01 |  | verapamil |  |
|  |  |  |  |  |
|  | **Diuretic (thiazide and thiazide-like)** | | |  |
|  | **ATC code** |  | **Generic name** |  |
|  | C03BA04 |  | chlortalidone |  |
|  | C03BA07 |  | clofenamide |  |
|  | C03BA03 |  | clopamide |  |
|  | C03AA03 |  | hydrochlorothiazide |  |
|  | C03AA02 |  | hydroflumethiazide |  |
|  | C03BA11 |  | indapamide |  |
|  | C03BA08 |  | metolazone |  |
|  | C03AA06 |  | trichlormethiazide |  |
|  |  |  |  |  |
|  | **Diuretic (non-thiazide)** | | |  |
|  | **ATC code** |  | **Generic name** |  |
|  | S01EC01 |  | acetazolamide |  |
|  | C03DB01 |  | amiloride |  |
|  | C03CA02 |  | bumetanide |  |
|  | C03DA04 |  | eplerenone |  |
|  | C03CC01 |  | etacrynic acid |  |
|  | C03CA01 |  | furosemide |  |
|  | C03DA01 |  | spironolactone |  |
|  | C03DB02 |  | triamterene |  |
|  |  |  |  |  |
|  | **α-blocker** |  |  |  |
|  | **ATC code** |  | **Generic name** |  |
|  | G04CA01 |  | alfuzosin |  |
|  | C02CA04 |  | doxazosin |  |
|  | C02CA01 |  | prazosin |  |
|  | G04CA04 |  | silodosin |  |
|  | G04CA02 |  | tamsulosin |  |
|  | G04CA03 |  | terazosin |  |
|  |  |  | **(To be continued)** |  |
|  |  |  |  |  |
| **(Continued)** | | |  |  |
|  | **β-blocker** |  |  |  |
|  | **ATC code** |  | **Generic name** |  |
|  | C07AB04 |  | acebutolol |  |
|  | C07AA01 |  | alprenolol |  |
|  | C07AB03 |  | atenolol |  |
|  | C07AB05 |  | betaxolol |  |
|  | C07AB07 |  | bisoprolol |  |
|  | C07AA15 |  | carteolol |  |
|  | C07AG02 |  | carvedilol |  |
|  | C07AG01 |  | labetalol |  |
|  | C07AB02 |  | metoprolol |  |
|  | C07AA12 |  | nadolol |  |
|  | C07AA02 |  | oxprenolol |  |
|  | C07AA03 |  | pindolol |  |
|  | C07AA05 |  | propranolol |  |
|  | C07AA07 |  | sotalol |  |
|  | C07AA06 |  | timolol |  |
|  |  |  |  |  |
|  | **Other blood pressure-lowering drug** | | |  |
|  | **ATC code** |  | **Generic name** |  |
|  | C09XA02 |  | aliskiren |  |
|  | C02KX02 |  | ambrisentan |  |
|  | C02KX01 |  | bosentan |  |
|  | C02AC01 |  | clonidine |  |
|  | C02CC02 |  | guanethidine |  |
|  | C02DB02 |  | hydralazine |  |
|  | C02AB01 |  | methyldopa (levorotatory) | |
|  | C02AB02 |  | methyldopa (racemic) | |
|  | C02DC01 |  | minoxidil |  |
|  | C02AA01 |  | rescinnamine |  |
|  | C02AA02 |  | reserpine |  |

Abbreviations: ATC, Anatomical Therapeutic Chemical.
